# Dietary patterns and hypertension among Chinese adults: a nationally representative cross-sectional study

**DOI:** 10.1186/1471-2458-11-925

**Published:** 2011-12-14

**Authors:** Dong Wang, Yuna He, Yanping Li, Dechun Luan, Xiaoguang Yang, Fengying Zhai, Guansheng Ma

**Affiliations:** 1 National Institute for Nutrition and Food Safety, Chinese Center for Disease Control and Prevention, 7 Pan Jia Yuan Nan Li, Chaoyang District, Beijing 100021, China; 2 Civil Aviation Medicine Center, Civil Aviation Administration of China, Chaoyang District, Beijing, China; 3 Liaoning Provincial Center for Disease Control and Prevention, Heping District, Shenyang, Liaoning Province, China

## Abstract

**Background:**

Several healthful dietary patterns appear to be effective at lowering blood pressure and preventing hypertension. However, the relationship between dietary patterns and hypertension among a representative Chinese population sample is unclear.

**Methods:**

A nationally representative sample of 23 671 participants aged 18-59 years were recruited by the 2002 China National Nutrition and Health Survey. All participants had their blood pressure measured with standardized mercury sphygmomanometers. Hypertension was defined as systolic blood pressure ≥140 mmHg and/or diastolic blood pressure ≥90 mmHg. We conducted factor analysis using dietary information from a validated food frequency questionnaire to derive dietary patterns. Information of participants on physical activities, education level, annual household income, smoking status and family history of hypertension was collected by interviewer-administrated questionnaires.

**Results:**

Three major dietary patterns, defined as 'Western', 'traditional northern', and 'traditional southern', were identified. Participants with the highest quartile for the score of the Western pattern had significantly higher blood pressure comparing with counterparts in the lowest quartile. In contrast, participants in the top quartile for the score of the traditional southern pattern presented significantly lower blood pressure comparing with counterparts in the lowest quartile. In multivariate analyses the traditional northern pattern score was associated with an odds ratio (OR) of 1.30 (95% confidence interval (CI) 1.11-1.53, *P *for trend = 0.0001) comparing with the lowest quartile. The OR for the top quartile of score for the traditional southern pattern was 0.73 (95% CI, 0.59-0.89, *P *for trend = 0.0040) compared with the lowest quartile of traditional southern pattern score. However, the significant association between the traditional northern pattern and prevalence of hypertension disappeared after further adjusting for body mass index (BMI) (*P *for trend = 0.3), whereas the association between the traditional southern pattern and prevalence of hypertension persisted after further adjusting for BMI (*P *for trend = 0.01).

**Conclusions:**

We observed a positive relationship between the traditional northern pattern and hypertension that was mediated through differences in BMI. In addition, the traditional southern pattern was significantly associated with lower odds of presenting with hypertension.

## Background

Suboptimal blood pressure is the number one attributable risk for death throughout the world [1] because of its major etiologic role in the development of cerebrovascular disease, ischemic heart disease, cardiac and renal failure, and cognitive impairment [2,3]. In China the prevalence of hypertension has increased by almost one third from 1991 to 2002 [4]. By 2002, 18% of the adults in China were hypertensive which equals to approximate 153 million individuals [5].

Some modifiable lifestyle risk factors for hypertension have been identified, including unhealthy dietary practice. To date, traditional analyses only examine the relationship between hypertension and a single or a small group of nutrients or foods [6-11]. This limited the application of their findings to hypertension prevention and treatment, since it oversimplified the regular diet [12]. Consequently, dietary-patterning analysis has been increasingly used as an alternative method to the traditional single nutrient analysis because it assesses interactive or synergistic effects of nutrients in the overall diet. Some observational studies and clinical trials among Western populations have suggested several healthful dietary patterns which appear to be effective at lowering blood pressure and preventing hypertension [13-19]. However, these dietary patterns were very different from common diets in China.

Previous studies have identified several dietary patterns and associated these dietary patterns with type 2 diabetes [20], anemia [21], and breast cancer [22] among several regionally representative samples in China. The Shanghai Men's Health Study (SMHS) reported [23] that a 'Fruit and Milk' pattern was associated with a lower prevalence of both pre-hypertension and hypertension among 39 252 Chinese men living in Shanghai. Our previous studies have identified four diverse dietary patterns based on the data from the 2002 China National Nutrition and Health Survey (2002 CNNHS) and associated these patterns with obesity [24], newly diagnosed glucose tolerance abnormalities [25] and stroke [26] among Chinese adults. However, little is known about the relationship between dietary patterns and hypertension among a representative Chinese population sample. We therefore investigated the associations between dietary patterns and newly diagnosed hypertension among a nationally representative Chinese adult population that participated in the 2002 CNNHS.

## Methods

### Study population

The 2002 CNNHS is a nationally representative cross-sectional study on nutrition and non-communicable chronic diseases. It covered all 31 provinces, autonomous regions, and municipalities directly under the Chinese central government (except Taiwan, Hong Kong, and Macao). A stratified, multistage probability cluster sampling design was used in this survey which has been described in detail previously [27]. On the basis of socioeconomic characteristics, the whole country was divided into six regions. As described elsewhere [4], in the first stage of sampling, 22 counties were randomly selected from each of the six regions in China. In the second stage, three townships were randomly selected from each of the selected counties. From each of these townships, two residential villages were randomly selected, and 90 households were then randomly sampled from each village for physical and medical examinations. One-third of all households were selected to participate in a dietary survey and blood draw. Ethics approval was obtained from the Ethics Committee of Chinese Center for Disease Control and Prevention. All participants gave informed consent. All subjects who were known to have hypertension, diabetes, dyslipidemia and stroke before the survey were excluded from the analyses (n = 1 466), because they may have changed their dietary habits to compensate their illness. In the study presented here, we surveyed 23 671 residents aged 18-59 years who were selected from the dietary assessments and physical examination as the population for analyses.

### Survey method

The physical examination involved the measurement of body height, weight and blood pressure. Body mass index (BMI) was calculated as weight (kg) divided by height (m) squared (kg/m^2^). Previous studies have described the blood pressure measurement in detail [4]. Briefly, all participants had their blood pressure measured with standardized mercury sphygmomanometers. Investigators were trained to perform blood pressure measurement and questionnaire before the survey. Two consecutive readings of blood pressure were taken on the right arm according to the 1999 World Health Organization/International Society of Hypertension guidelines on hypertension [2] with the participant in a seated position after 5 min of rest; the mean of the 2 measures was used for analysis. Hypertension was defined according to the Chinese Guidelines on Prevention and Control of Hypertension [28] and the Seventh Joint National Committee on Prevention, Detection, Evaluation, and Treatment of High Blood Pressure guidelines [29] as systolic blood pressure (SBP) ≥140 mmHg and/or diastolic blood pressure (DBP) ≥90 mmHg.

### Dietary assessment

A validated semiquantitative food frequency questionnaire (FFQ) was used to assess dietary intake over the period of one year prior to the study. We evaluated the relative validity of intake of food [30] and nutrients [31] derived from an FFQ among 23 198 participants (Men: 11 107, Women: 12 091) in the 2002 CNNHS. The estimates of food consumption and nutrient intake from the FFQ were compared with those based on 3-day weighed food records by relative under-reporting or over-reporting rate and spearman regression coefficients. We concluded that the FFQ appeared to be reasonably valid in the assessment of both food consumption and nutrient intake of Chinese adults. The FFQ includes 33 food items. For each food item or food group, subjects were asked the frequency of consumption (daily, weekly, monthly, yearly or never) and the amount of consumption in the unit of Liang (1 Liang = 50 g) per unit of time over the past 12 months. According to the similarity of nutrient profiles and culinary usage among the foods and the grouping scheme used in other studies, we collapsed the 33 food items into 23 predefined food groups (Additional file 1). Alcohol consumption was calculated based on the drinking frequency, and consumption of different types of liquor which was collected by FFQ also.

### Ascertainment of non-dietary factors

Information on physical activities was collected by trained investigators using a validated 1-year physical activity questionnaire [32]. The frequency and duration of the five categories of physical activities were recorded, including occupational physical activities, leisure time exercises, physical activities for transportation purposes, household work related activities, and sedentary activities (watching television, using computer, playing video games and reading during leisure time). The intensity of each activity in the questionnaire was coded according to the compendium of physical activities [33].

Physical activity level (PAL) was calculated by an equation recommended by the Institute of Medicine (IOM) [34] and was classified into four categories as shown below [32]:

▪ Sedentary: PAL 1.00-1.39;

▪ Low active: PAL 1.40-1.59;

▪ Active: PAL 1.60-1.89;

▪ Very active: PAL 1.90-2.50.

Participants' education level, annual household income, and family history of hypertension (in first degree relatives and grandparents) were collected by using the general information questionnaire. Participants' information on smoking habits was also obtained by a standardized questionnaire. We categorized the smoking status into "Never smoked", "Former smoker (A participant who had smoked at least 1 cigarette/day for more than 6 months (including 6 months) in his/her lifetime but he/she had quit smoking subsequently for more than 2 years when doing the interview)", "Current smoker (1-14 cigarettes/day)", and "Current smoker (≥15 cigarettes/day)". Participants responded to standardized questionnaires and the questionnaires were examined by trained investigators.

### Statistical analyses

Factor analysis (principal component) was used to derive food patterns based on the 23 food groups from the FFQ. We conducted the analysis using the FACTOR PROCEDURE in SAS (Statistical Analysis System, SAS Institute, Cary, NC, USA). The factors were rotated by an orthogonal transformation (Varimax rotation function in SAS) to achieve a more simplistic structure with greater interpretability. In considering the number of factors to retain, we evaluated eigenvalues (> 1), the Scree test, and the interpretability of the factors to determine which set of factors can most meaningfully describe distinct food patterns. Items were retained in a factor if they had an absolute correlation ≥ 0.30 with that factor. Factor scores for each pattern were calculated as the sum of the products of the factor loading coefficients and the standardized daily intake of each food associated with that pattern (Additional file 1).

Study participants were categorized into quartiles of dietary factor scores for each dietary pattern. Mean levels of quantitative variables were estimated using PROC SURVEYMEANS procedure of SAS for Windows 9.2 (SAS Institute Inc, Cary, NC). Survey weights were derived from the 2000 Census and associated administrative data [4]. PROC SURVEYFREQ procedure was used to obtain the frequencies for qualitative variables over all groups and within subgroups. Multivariate logistic regression, applied with PROC SURVEYLOGISTIC, was used to estimate the association between each dietary pattern and prevalence of hypertension, adjusting for age (continuous), sex (men/women), living area (urban/rural), education level (uneducated/primary school/middle school/higher education), presence or absence of a family history of hypertension, annual household income per family member (<800/800-1999/2000-4999/≥5000 RMB), alcohol consumption (continuous), total energy intake (continuous), PAL (sedentary/low active/active/very active), and smoking status (never smoked/former smoker/1-14 cigarettes/day/≥15 cigarettes/day). Since numerous studies [4,28,29] have indicated the strong relation between BMI and hypertension, we further included BMI in the model to assess whether the associations between dietary patterns and hypertension were mediated by weight status. Adjusted mean values of blood pressure associated with each category of dietary factor scores were compared using the PROC SURVEYREG procedure with adjustment for the covariates mentioned above. To address the possibility of misclassification bias, we conducted a sensitivity analysis by applying the cut-off points for stage 2 hypertension as systolic blood pressure ≥160 mm Hg and/or diastolic blood pressure ≥100 mm Hg [29]. All *P *values were 2-tailed (α = 0.05).

## Results

Three major dietary patterns were identified by the factor analysis (Additional file 1), which explained 28.2% of the variance. Factor-loading matrixes for the 3 dietary patterns are listed in the Additional file 1.

We observed evidence of westernization of the factor 1 through the food items (beef, lamb, dairy products, soft beverage, cake, and juice) and named it as 'Western pattern'. The traditional northern pattern (Factor 2), was characterized by high intakes of wheat flour products and starchy tubers, combined with low consumption of protein products such as pork, beef, poultry, aquatic products, or milk and milk products and represented a typical traditional diet in north China. The third factor, the traditional southern pattern, represented a typical traditional diet in south China, characterized by high intakes of fruit, pork, poultry, rice, vegetables, aquatic products and nuts.

Table 1 shows the selected characteristics of all participants according to the 3 dietary patterns. The mean age of the study population was 39.8 years. Of the study population 53.0% were men. Participants that reported a family history of hypertension amounted to 15.2%. The mean values of SBP and DBP for the whole study group were 118.8 mmHg and 77.5 mmHg, respectively. The newly diagnosed prevalence of hypertension among the study population was 29.4%. Individuals with high Western pattern score were younger, had higher education level, higher income, and lower intake of sodium compared to those individuals with lower Western pattern score. Most of the participants with higher traditional northern pattern score were from rural area, with lower income, higher blood pressure and higher intake of sodium compared to the lower traditional northern pattern score group. Participants with higher traditional southern pattern score were found to be more likely to consume alcohol and tobacco and engage in active physical activities compared to their counterparts with lower traditional southern pattern score.

**Table 1:** Selected characteristics of Chinese adults according to different dietary patterns^1^

	All	Western pattern		Traditional northern pattern		Traditional southern pattern	
		
		Q1	Q4	Q1	Q4	Q1	Q4
Age (years)	39.8 (0.1)	40.7 (0.2)	37.6 (0.3)	41.1 (0.2)	39.6 (0.2)	40.0 (0.2)	38.7 (0.2)

Body mass index (kg/m^2^)	22.7 (0.0)	22.4 (0.1)	23.0 (0.1)	21.9 (0.1)	23.2 (0.1)	22.7 (0.1)	23.1 (0.1)

Systolic blood pressure (mmHg)	118.8 (0.2)	118.6 (0.4)	118.1 (0.5)	119.0 (0.4)	120.5 (0.3)	118.6 (0.5)	118.0 (0.4)

Diastolic blood pressure (mmHg)	77.5 (0.1)	77.1 (0.3)	77.6 (0.4)	76.6 (0.2)	78.9 (0.2)	77.6 (0.3)	77.6 (0.2)

Total energy intake (kcal)	2476.4 (7.4)	2408.2 (17.2)	2524.5 (17.8)	2685.6 (13.5)	2727.3 (15.3)	2026.0 (15.9)	2894.0 (15.1)

Sodium intake (mg)	6099.9 (25.7)	6196.6 (64.2)	5841.0 (58.4)	5895.0 (48.0)	6557.6 (55.1)	5873.3 (72.3)	6491.5 (62.2)

Alcohol consumption (g/day)	0.8 (0.0)	0.8 (0.0)	0.9 (0.0)	0.7 (0.0)	0.9 (0.0)	0.5 (0.0)	1.0 (0.0)

Sex (% of men)	53.0 (0.6)	51.8 (1.3)	56.5 (1.3)	54.6 (1.2)	59.4 (1.0)	49.1 (1.4)	61.5 (1.0)

Prevalence of hypertension^2 ^(%)	29.4 (0.6)	28.1 (1.1)	28.7 (1.3)	27.5 (1.1)	33.2 (1.0)	29.5 (1.3)	27.1 (1.0)

Family history (%)	15.2 (0.4)	12.4 (0.9)	21.9 (1.0)	10.4 (0.6)	17.4 (0.8)	14.4 (1.1)	20.2 (0.9)

Living area (%)							

Urban area	27.5 (0.0)	29.5 (0.5)	51.1 (1.0)	10.4 (0.5)	16.7 (0.9)	33.5 (0.6)	38.3 (0.9)

Rural area	72.5 (0.0)	70.5 (0.5)	48.9 (1.0)	89.6 (0.5)	83.3 (0.9)	66.5 (0.6)	61.7 (0.9)

Annual income per family member (RMB)							

<800	12.1 (0.3)	15.2 (0.7)	6.1 (0.7)	11.7 (0.8)	17.1 (0.7)	18.9 (0.6)	5.3 (0.6)

800-1999	32.8 (0.5)	33.9 (0.9)	20.4 (1.2)	40.5 (1.1)	40.9 (0.9)	34.0 (0.9)	21.4 (0.9)

2000-4999	31.4 (0.5)	28.7 (1.0)	28.4 (1.2)	34.2 (1.1)	30.8 (1.0)	23.5 (1.1)	35.7 (1.0)

≥5000	23.7 (0.4)	22.2 (0.9)	45.2 (1.2)	13.6 (0.6)	11.2 (0.7)	23.5 (1.0)	37.6 (1.0)

Education level (%)							

Uneducated	36.6 (0.4)	41.3 (0.9)	20.0 (1.1)	52.7 (1.1)	36.6 (0.9)	37.3 (0.9)	24.1 (0.9)

Primary school	38.2 (0.6)	35.3 (1.2)	35.5 (1.3)	36.1 (1.0)	44.2 (1.0)	36.2 (1.3)	40.8 (1.1)

Middle school	18.2 (0.5)	17.2 (1.1)	28.2 (1.2)	9.9 (0.8)	15.4 (0.8)	19.6 (1.3)	23.1 (0.9)

Higher education	7.0 (0.3)	6.1 (0.7)	16.3 (0.9)	1.2 (0.2)	3.7 (0.4)	7.0 (0.9)	12.0 (0.7)

Physical activity level (%)							

Sedentary	51.6 (0.5)	53.7 (1.0)	36.7 (1.3)	69.1 (0.9)	53.6 (1.0)	49.0 (1.0)	44.1 (1.0)

Low active	21.6 (0.5)	21.3 (1.1)	17.6 (0.9)	19.1 (0.7)	23.5 (0.8)	23.9 (1.2)	18.1 (0.8)

Active	13.8 (0.4)	13.0 (1.0)	21.9 (1.0)	6.5 (0.5)	12.2 (0.7)	14.2 (1.1)	18.4 (0.8)

Very active	13.0 (0.4)	11.9 (0.9)	23.8 (1.1)	5.3 (0.4)	10.7 (0.8)	12.8 (1.1)	19.4 (0.9)

Smoking status							

Never smoked	71.1 (0.5)	73.6 (1.1)	69.1 (1.2)	70.1 (1.1)	66.2 (1.0)	75.3 (1.2)	65.1 (1.1)

Former smoker	1.5 (0.1)	1.5 (0.3)	1.8 (0.3)	1.6 (0.3)	1.5 (0.2)	1.8 (0.4)	2.3 (0.3)

1-14 cigarettes/day	10.6 (0.4)	9.5 (0.8)	12.9 (0.9)	8.3 (0.5)	13.9 (0.8)	10.6 (0.9)	12.6 (0.8)
≥15 cigarettes/day	16.7 (0.4)	15.4 (0.9)	16.2 (0.9)	19.9 (1.0)	18.4 (0.8)	12.4 (0.8)	20.0 (0.9)

^1^Values are means (standard error) for the quantitative variables or proportions (standard error) for the qualitative variables

^2^Hypertension was defined as systolic blood pressure ≥140 mmHg and/or diastolic blood pressure ≥90 mmHg

After adjusting for age, sex, living area, education level, family history of hypertension, income, alcohol consumption, total energy intake, PAL and smoking status the adjusted mean value of SBP was 1.4 mmHg higher and DBP was 1.9 mmHg higher for participants in the highest quartile of scores for the traditional northern pattern compared that for participants in the lowest quartile (Table 2). Participants with the highest quartile of scores for the traditional southern pattern had 0.8 mmHg lower SBP and 0.8 mmHg lower DBP, compared with counterparts in the lowest quartile of scores for the traditional southern pattern. The adjusted mean SBP was 1.2 mmHg higher and DBP was 0.5 mmHg higher among participants in the fourth quartile of scores for the Western pattern than that among participants in the first quartile. After further adjustment for BMI, the association between blood pressure and dietary pattern was appreciably attenuated (Table 2).

**Table 2:** Blood pressures according to the quartiles of factor score for each dietary pattern^1^

	Quartiles of factor score				*P *for trend
		
	Q1	Q2	Q3	Q4	
Systolic blood pressure					

Western pattern					

Model 1	118.2 (0.4)	118.9 (0.3)	119.4 (0.4)	119.2 (0.5)	0.1340

Model 2	116.8 (0.2)	117.8 (0.2)	118.3 (0.3)	118.0 (0.3)	<.0001

Model 3	117.1 (0.2)	117.9 (0.2)	118.1 (0.2)	118.2 (0.3)	0.0002

Traditional northern pattern					

Model 1	118.2 (0.4)	118.0 (0.5)	119.3 (0.4)	120.4 (0.3)	<.0001

Model 2	117.5 (0.2)	116.6 (0.2)	118.0 (0.3)	118.9 (0.3)	<.0001

Model 3	118.4 (0.2)	117.0 (0.2)	117.6 (0.3)	118.3 (0.3)	<.0001

Traditional southern pattern					

Model 1	118.7 (0.5)	119.1 (0.4)	119.3 (0.4)	118.3 (0.3)	0.2638

Model 2	117.7 (0.3)	118.3 (0.2)	118.1 (0.2)	116.9 (0.3)	0.0002

Model 3	117.4 (0.3)	118.4 (0.2)	118.4 (0.2)	117.0 (0.2)	<.0001

Diastolic blood pressure					

Western pattern					

Model 1	76.9 (0.3)	77.3 (0.2)	77.9 (0.3)	78.2 (0.3)	0.0069

Model 2	76.2 (0.1)	77.1 (0.1)	77.2 (0.2)	76.7 (0.2)	<.0001

Model 3	76.5 (0.1)	77.2 (0.1)	77.0 (0.2)	76.8 (0.2)	0.0007

Traditional northern pattern					

Model 1	76.2 (0.2)	77.1 (0.3)	78.5 (0.3)	78.8 (0.2)	<.0001

Model 2	76.2 (0.1)	75.9 (0.1)	77.3 (0.2)	78.1 (0.2)	<.0001

Model 3	76.8 (0.1)	76.1 (0.1)	77.0 (0.2)	77.6 (0.2)	<.0001

Traditional southern pattern					

Model 1	77.7 (0.3)	77.3 (0.3)	77.6 (0.3)	77.6 (0.2)	0.7412

Model 2	77.2 (0.2)	76.8 (0.2)	76.9 (0.2)	76.4 (0.2)	0.0703

Model 3	77.0 (0.2)	76.9 (0.1)	77.1 (0.2)	76.5 (0.2)	0.0817

^1^Values are adjusted means (standard error)

Model 1: adjusted for age (continuous) and sex (men/women)

Model 2: model 1 additionally adjusted for living area (urban/rural), education level (uneducated/primary school/middle school/higher education), presence or absence of a family history of hypertension, annual household income per family member (<800/800-1999/2000-4999/≥5000 RMB), alcohol consumption (continuous), total energy intake (continuous), physical activity level (sedentary/low active/active/very active), and smoking status (never smoked/former smoker/1-14 cigarettes/day/≥15 cigarettes/day)

Model 3: model 2 additionally adjusted for body mass index (continuous)

Table 3 presents association of each dietary pattern with the newly diagnosed prevalence of hypertension. After adjustment for age and sex, a higher traditional northern pattern score was associated with a higher prevalence of hypertension (*P *for trend < 0.0001). The OR comparing the highest traditional northern pattern quartile to the lowest was 1.44 (95% CI, 1.24-1.67). After further adjusting for living area, education level, family history of hypertension, income, PAL, alcohol consumption, total energy intake, and smoking status the association between the traditional northern pattern and hypertension was attenuated but remained significant and positive (*P *for trend = 0.0001). The OR for the top quartile was 1.30 (95% 1.11-1.53) compared with the lowest quartile of traditional northern pattern score. However, the significant association between the traditional northern pattern and prevalence of hypertension disappeared after further adjusting for BMI (*P *for trend = 0.3). A higher traditional southern pattern score was associated with a lower prevalence of hypertension after adjusting age, sex, living area, education level, family history of hypertension, income, PAL, alcohol consumption, total energy intake and smoking status (*P *for trend = 0.004). The inverse association remained significant after further adjustment for BMI (*P *for trend = 0.01). The OR for the top quartile was 0.75 (95% CI, 0.60-0.92) compared with the lowest quartile of traditional southern pattern score. After adjusting for age and sex a positive association was observed between the Western pattern score and hypertension (*P *for trend < 0.0001). However, the association disappeared after further multivariate adjustment. The associations between dietary patterns and prevalence of hypertension were quite consistent in men and women (Additional file 2).

**Table 3:** Association of dietary patterns with newly diagnosed hypertension^1^

	Quartiles of factor score				*P *for trend
		
	Q1	Q2	Q3	Q4	
Western pattern					

Model 1	Reference	1.10 (0.95-1.27)	1.24 (1.06-1.45)	1.21 (1.02-1.44)	<.0001

Model 2	Reference	1.15 (0.99-1.34)	1.20 (1.01-1.42)	1.07 (0.86-1.33)	0.3759

Model 3	Reference	1.12 (0.96-1.31)	1.11 (0.93-1.32)	1.04 (0.83-1.31)	0.6638

Traditional northern pattern					

Model 1	Reference	1.13 (0.96-1.34)	1.41 (1.20-1.65)	1.44 (1.24-1.67)	<.0001

Model 2	Reference	0.97 (0.81-1.16)	1.22 (1.03-1.44)	1.30 (1.11-1.53)	0.0001

Model 3	Reference	0.90 (0.75-1.09)	1.02 (0.86-1.22)	1.06 (0.90-1.24)	0.3341

Traditional southern pattern					

Model 1	Reference	1.00 (0.85-1.17)	1.05 (0.89-1.23)	0.89 (0.76-1.04)	0.2850

Model 2	Reference	0.97 (0.81-1.16)	0.98 (0.80-1.18)	0.73 (0.59-0.89)	0.0040

Model 3	Reference	1.02 (0.84-1.23)	1.04 (0.85-1.28)	0.75 (0.60-0.92)	0.0122

^1^Values are odds ratios (95% confidence interval)

Model 1: adjusted for age (continuous) and sex (men/women)

Model 2: model 1 additionally adjusted for living area (urban/rural), education level (uneducated/primary school/middle school/higher education), presence or absence of a family history of hypertension, annual household income per family member (<800/800-1999/2000-4999/≥5000 RMB), alcohol consumption (continuous), total energy intake (continuous), physical activity level (sedentary/low active/active/very active), and smoking status (never smoked/former smoker/1-14 cigarettes/day/≥15 cigarettes/day)

Model 3: model 2 additionally adjusted for body mass index (continuous)

After applying the sensitivity analysis, the results did not materially change. The association between the traditional northern pattern and hypertension was even stronger after applying the stage 2 hypertension cut-off points., A higher traditional northern pattern score was associated with a higher prevalence of hypertension (*P *for trend < 0.0001). The multivariable-adjusted OR for the top quartile was 1.63 (95% CI, 1.18-2.26) compared with the lowest quartile of traditional northern pattern score. After further adjustment for BMI the OR comparing the highest traditional northern pattern quartile to the lowest was 1.26 (95% CI, 0.90-1.76).

## Discussion

In this study, we identified three major dietary patterns at a national level and associated them with hypertension. The traditional northern pattern was associated with both higher prevalence of hypertension and blood pressure, but the associations seemed to be largely explained by BMI. Participants with higher score for the traditional southern pattern have significantly lower prevalence of hypertension and blood pressure. This association was independent of BMI.

Previous studies [35,36] have reported higher prevalence of hypertension and higher blood pressure level in the northern than in the southern areas of China. Zhao et al. [36] found significantly higher hypertension prevalence for the north (22.3%) than for the south (7.2%) and suggested that the north-south differences in hypertension were mainly explained by the dietary factors. Our results were consistent with the previous observations. The traditional southern pattern was related to the lower prevalence of hypertension and blood pressure, whereas the traditional northern pattern was associated with higher prevalence of hypertension and blood pressure. The differences in actual blood pressure levels in this study were small which raises the concern about clinical relevance. However, as Cook et al. [37,38] have suggested, on a population level even apparently minor differences in blood pressure could have major effects for these highly prevalent diseases. In their study, a 2 mmHg reduction in diastolic blood pressure could result in 17% decrease in the prevalence of hypertension as well as 6% and 15% reduction in the risk of coronary heart disease and stroke respectively. Additionally, because we have excluded all the subjects who had known their hypertension status, the mean difference of blood pressure we observed was more likely to be underestimated.

It remains unclear which dietary factor in the traditional northern pattern may contribute to the detrimental effects on hypertension. Even the traditional northern pattern adopters consumed a limited amount of animal source food they seem very vulnerable to hypertension. High intake of sodium [39-41] in this pattern may be associated with hypertension. Accordingly, we further adjusted sodium intake in the multi-variable logistic models. However, the correlation between the traditional northern pattern and the prevalence of hypertension after further adjustment was still significant, only slightly attenuated, which indicated that sodium intake only partially explained the association. Notably, patterns of high intake of refined carbohydrates and low consumption of fresh vegetables, fruits and aquatic products may also contribute to the development of hypertension among this population. Support for this postulation comes from considerable evidence reported by previous studies [16,42,43]. Similar diet pattern was also observed in other East Asian countries. In Korea, Kim et al. [44] found adaptors of the traditional Korean dietary pattern in which wheat is also consumed as the staple portion in a meal did not show significantly lower blood pressure. Kim suggested this phenomenon might be partially explained by the dietary practice of Koreans who consumed salted vegetables instead of fresh vegetables. It is noteworthy that the traditional northern pattern adopters also tended to be physically inactive as shown in Table 1, which may relate to the strong mediating role of BMI in explaining the association between the traditional northern pattern and hypertension. Furthermore, further research may be conducted to investigate the low physical activity level in this population, which has the great potential to suggest a more feasible intervention point than requesting a change in their traditional northern diet.

In the present study, the inverse and independent of BMI association between the traditional southern pattern and hypertension might be explained by the high consumption of vegetables, fruits, aquatic products, soybean products and nuts. These protective food items have been emphasized by other dietary patterns [16-19,45-48] which have been proven to lower blood pressure and prevent hypertension. Similar dietary patterns have been reported by study in South China [21] and our previous studies [24-26]. The traditional southern pattern also shares most of food items which included in other typical oriental dietary patterns described by multi-center international studies [49] and studies in East Asia [50].The inverse association between the traditional southern pattern score and prevalence of hypertension is further supported by a study focused on a traditional Japanese diet. Here individuals adopted a traditional Japanese dietary pattern, which is characterized by higher intake of soybean products, fish, seaweeds, vegetables, fruits, pork and poultry. Those following this dietary pattern had lower SBP, DBP, and pulse pressure compared to the Western dietary pattern adopters [50]. In the SMHS [23], a 'fruit and milk' pattern also showed strong protective association for blood pressure and prevalence of hypertension. However, our traditional southern pattern is somewhat different in terms of pattern structure from the SMHS's 'fruit and milk' pattern. The traditional southern pattern contains a diverse dietary portfolio (fruit, poultry, pork, aquatic product, soybean product, vegetables and nuts), while the 'fruit and milk' is loaded heavily only on fruit and milk.

Previous studies have associated a Western dietary pattern (high in red meat, processed meat, refined grains, sweets and deserts, French fries, and high-fat dairy products) with a significantly increased risk of hypertension in the Western populations [21,51,52]. We observed Western-style changes in dietary habits of the Western pattern adopters in the present study. For instance, they tend to take more meat and drink high-sugar beverages, instead of the traditional cereal based, low-fat and high vegetable content stir-fried meals. This pattern had broken away from the traditional Chinese food culture and was also related with higher blood pressure. However, the association between the Western pattern and blood pressure in the present study was not as strong as that observed in the Western populations. Even this pattern was not related to the prevalence of hypertension. This may be partly due to the Western pattern adopters also consumed high levels of fruit and aquatic products which are protective for hypertension.

Potential limitations of our study should not be overlooked. By using a cross-sectional study we cannot formally draw conclusions about causality. However, it should be noted that when dietary patterns are investigated, it is unlikely that reverse causality has played a role. Furthermore, this problem is mitigated by the exclusion of patients who were known to have hypertension, diabetes, dyslipidemia and stroke prior to the survey from the analyses, although residual bias may still exist. Data on dietary intake were collected by questionnaires referring to a period of one year. However, dietary habits may change over a lifetime, and these changes may have an additional impact on hypertension. In addition, lack of detailed information on foods (e.g., types of meats) also made it difficult to characterize dietary patterns in more detail. Although we have adjusted for major sociodemographic characteristics and lifestyle factors simultaneously, residual confounding by unknown or unmeasured factors may be present. Conversely, given the magnitude of many of the risk estimates and consistency of our results with prior controlled trials and cohort studies of some dietary patterns, it is improbable that all of the observed risk differences are attributed to the results of residual confounding. Dichotimization of some sociodemogrphic and lifestyle controlled variables (such as living area) that have graded effects on risk also could have attenuated the magnitude of association compared with other surveys. Given that blood pressure readings taken on one occasion, it is possible that some people may have a transient rise in blood pressure produced by the interviews, which create a potential for misclassification bias. However, we have included a sensitivity analysis by applying higher cut-off points for stage 2 hypertension to address the possibility of misclassification bias and observed no materially changed results after the sensitivity analysis. At last, since the definition of former smoker in this study is a participant who had smoked at least 1 cigarette/day for more than 6 months (including 6 months) in his/her lifetime but he/she had quit smoking subsequently for more than 2 years when doing the interview, it is possible that participants who had quit for less than two years were classified as current smoker. This may have an effect of biasing the association between current smoking and hypertension towards null. However, previous studies have reported short-term smoking cessation (eg. Less than 2 years) may not significantly influence on risk of hypertension [53] and alleviating arterial stiffness [54]. Additionally, According to a national representative survey [55] conducted in China in 1996, of all the subjects who had tried to quit smoking less than 4% had abstained from smoking for at least 2 years. Therefore, it is unlikely that the association between dietary pattern and hypertension could be substantially changed by this misclassification.

## Conclusions

In conclusion, major dietary patterns identified by means of factor analysis significantly associated with the prevalence of hypertension among Chinese adults, provides sound evidence for specific diet intervention strategies for individuals with different dietary patterns. Considering the high prevalence (29.4%) of undiagnosed hypertension revealed by the present study, China needs to strengthen nation-wide early diagnosis capability and encourage integrated primary prevention measures to combat the growing disease burden.

## Competing interests

The authors declare that they have no competing interests.

## Authors' contributions

DW had full access to all of the data in the study. DW takes responsibility for the integrity of the data and the accuracy of the data analysis. XY, FZ and GM were the main investigators of 2002CNNHS. DW, YH, YL and GM contributed to the discussion and interpretation of the data, and to the writing of the manuscript. The present study was conceptualized by DW and GM. GM supervised the study. All authors read and approved the final manuscript.

## Pre-publication history

The pre-publication history for this paper can be accessed here:

http://www.biomedcentral.com/1471-2458/11/925/prepub

## Supplementary Material

Additional file 1 **Factor loading for each dietary pattern among Chinese adults**. Click here for file

Additional file 2 **Association of dietary patterns with newly diagnosed hypertension stratified by sex**. Click here for file
