# Supplementary material for: Dietary patterns and hypertension among Chinese adults: a nationally representative cross-sectional study
Source: BMC Public Health. 2011 Dec 14;11:925. doi: 10.1186/1471-2458-11-925 (PMC3299712; doi:10.1186/1471-2458-11-925)
Supplement: Additional file 1 — Factor loading for each dietary pattern among Chinese adults. [file 1471-2458-11-925-S1.DOC]

| Additional file 1 - Factor loading for each dietary pattern among Chinese adults. | | | |
| --- | --- | --- | --- |
|  | Factor 1:  Western pattern | Factor 2:  Traditional northern pattern | Factor 3:  Traditional southern pattern |
| Rice and rice products | -0.20014 | **-0.73158** | 0.15901 |
| Wheat and wheat products | 0.10097 | **0.74829** | 0.00511 |
| Other cereals | -0.10630 | **0.57093** | 0.00259 |
| Starchy tubers | -0.07981 | **0.50782** | 0.10170 |
| Fried wheat products | 0.06580 | **0.37881** | 0.27534 |
| Pork | 0.10060 | -0.28253 | **0.53980** |
| Beef/lamb | **0.55658** | 0.11846 | 0.25952 |
| Poultry | 0.27910 | -0.17860 | **0.56600** |
| Animal giblets | 0.27611 | -0.07772 | **0.39305** |
| Aquatic product | 0.20757 | -0.28338 | **0.49192** |
| Milk | **0.57600** | 0.07084 | 0.23208 |
| Milk powder | 0.18795 | 0.07401 | 0.17869 |
| Cheese | **0.45273** | 0.10504 | 0.00624 |
| Yogurt | **0.56387** | 0.06314 | 0.13424 |
| Egg | 0.05199 | 0.29939 | **0.50470** |
| Soybean products | -0.12624 | 0.08190 | **0.44417** |
| Dry bean | 0.14661 | 0.06351 | 0.09336 |
| Vegetables | -0.18052 | **-0.30157** | **0.37347** |
| Cake | **0.33848** | 0.00003 | **0.39700** |
| Fruit | 0.26226 | 0.13832 | **0.57519** |
| Nuts | 0.18424 | 0.06605 | **0.32087** |
| Juice | **0.42415** | -0.04063 | 0.25197 |
| Other beverages | **0.57180** | 0.03330 | 0.02984 |

Absolute values ≥0.30 were in bold.
