# Supplementary material for: Dietary patterns and hypertension among Chinese adults: a nationally representative cross-sectional study
Source: BMC Public Health. 2011 Dec 14;11:925. doi: 10.1186/1471-2458-11-925 (PMC3299712; doi:10.1186/1471-2458-11-925)
Supplement: Additional file 2 — Association of dietary patterns with newly diagnosed hypertension stratified by sex. [file 1471-2458-11-925-S2.DOC]

### Additional file 2 Association of dietary patterns with newly diagnosed hypertension stratified by sex^1^

|  | Factor score | | | | *P* for trend | *P* for interaction  sex*dietary pattern |
| --- | --- | --- | --- | --- | --- | --- |
|  | Q1 | Q2 | Q3 | Q4 |
| Western pattern | |  |  |  |  |  |
| Model 1 |  |  |  |  |  |  |
| Men | Reference | 1.05 (0.88-1.27) | 1.30 (1.06-1.59) | 1.26 (1.01-1.57) | 0.0102 | 0.1290 |
| Women | Reference | 1.17 (0.93-1.47) | 1.17 (0.93-1.47) | 1.15 (0.88-1.49) | 0.2317 |
| Model 2 |  |  |  |  |  |  |
| Men | Reference | 1.11 (0.91-1.35) | 1.24 (0.99-1.56) | 1.05 (0.80-1.37) | 0.5091 | 0.1106 |
| Women | Reference | 1.25 (0.98-1.59) | 1.14 (0.89-1.46) | 1.12 (0.80-1.59) | 0.4889 |
| Model 3 |  |  |  |  |  |  |
| Men | Reference | 1.07 (0.88-1.30) | 1.13 (0.89-1.43) | 0.97 (0.74-1.28) | 0.9851 | 0.5623 |
| Women | Reference | 1.23 (0.96-1.57) | 1.08 (0.84-1.39) | 1.17 (0.80-1.70) | 0.5119 |
| Traditional northern pattern | |  |  |  |  |  |
| Model 1 |  |  |  |  |  |  |
| Men | Reference | 1.40 (1.12-1.75) | 1.74 (1.41-2.15) | 1.56 (1.29-1.90) | <.0001 | 0.0614 |
| Women | Reference | 0.84 (0.66-1.08) | 1.06 (0.85-1.34) | 1.29 (1.02-1.62) | 0.0154 |
| Model 2 |  |  |  |  |  |  |
| Men | Reference | 1.14 (0.90-1.45) | 1.50 (1.20-1.87) | 1.43 (1.17-1.75) | <.0001 | 0.0229 |
| Women | Reference | 0.77 (0.59-1.01) | 0.92 (0.71-1.18) | 1.13 (0.88-1.44) | 0.3263 |
| Model 3 |  |  |  |  |  |  |
| Men | Reference | 1.07 (0.83-1.38) | 1.28 (1.02-1.61) | 1.20 (0.97-1.48) | 0.0352 | 0.0073 |
| Women | Reference | 0.72 (0.55-0.94) | 0.76 (0.59-0.98) | 0.88 (0.69-1.12) | 0.2555 |
| Traditional southern pattern | |  |  |  |  |  |
| Model 1 |  |  |  |  |  |  |
| Men | Reference | 0.88 (0.71-1.10) | 1.06 (0.85-1.31) | 0.88 (0.72-1.08) | 0.5310 | 0.4930 |
| Women | Reference | 0.88 (0.69-1.13) | 1.02 (0.80-1.31) | 1.17 (0.92-1.47) | 0.2940 |
| Model 2 |  |  |  |  |  |  |
| Men | Reference | 0.87 (0.68-1.10) | 0.98 (0.77-1.25) | 0.74 (0.57-0.95) | 0.0591 | 0.3223 |
| Women | Reference | 1.09 (0.83-1.42) | 0.94 (0.69-1.28) | 0.64 (0.46-0.91) | 0.0107 |
| Model 3 |  |  |  |  |  |  |
| Men | Reference | 0.87 (0.68-1.11) | 1.01 (0.78-1.29) | 0.74 (0.57-0.96) | 0.0729 | 0.7575 |
| Women | Reference | 1.18 (0.88-1.56) | 1.04 (0.76-1.44) | 0.67 (0.47-0.96) | 0.0333 |

^1^ Values are odds ratios (95% confidence interval).

Model 1: adjusted for age (continuous) and sex (men/women).

Model 2: model 1 additionally adjusted for living area (urban/rural), education level (uneducated/primary school/middle school/higher education), presence or absence of a family history of hypertension, annual household income per family member (＜800/800-1999/2000-4999/≥5000 RMB), alcohol consumption (continuous), total energy intake (continuous), physical activity level (sedentary/low active/active/very active), and smoking status (never smoked/former smoker/1-14 cigarettes/day/≥15 cigarettes/day).

Model 3: model 2 additionally adjusted for body mass index (continuous).
